# Evaluation of sexual minority identity as a moderator of the association between intimate partner violence and suicidal ideation and attempts among a national sample of youth

**DOI:** 10.1371/journal.pone.0236880

**Published:** 2020-08-07

**Authors:** Liesl A. Nydegger, Lyzette Blanco, C. Nathan Marti, Daniel Kreitzberg, Katherine Quinn

**Affiliations:** 1 Health Behavior and Health Education, Department of Kinesiology and Health Education, The University of Texas at Austin, Austin, TX, United States of America; 2 Edith Neumann School of Health and Human Services, Department of Health Science, Touro University Worldwide, Los Alamitos, CA, United States of America; 3 Abacist Analytics, LLC, Austin, TX, United States of America; 4 Health Behavior and Health Education, Department of Kinesiology and Health Education, The University of Texas at Austin, Austin, TX, United States of America; 5 Center for AIDS Intervention Research, Department of Psychiatry, Medical College of Wisconsin, Milwaukee, WI, United States of America; University of Westminster, UNITED KINGDOM

## Abstract

Sexual minority (SM) youth are at high risk for intimate partner violence (IPV) and suicidal ideation/attempts compared to their heterosexual peers. We examined whether SM identity enhanced the relationship between experiences of IPV and suicidal ideation/attempts. Weighted logistic regression models were run using the 2017 Youth Risk Behavior Survey. All main effects were significant; each SM identity and both physical and sexual IPV were significantly associated with suicidal ideation and suicide attempts. The interaction between bisexual identity and physical IPV was significant for suicidal ideation; as physical IPV experiences increased, the difference between bisexual identity and heterosexual youth was non-significant. Findings suggest exploring trauma and suicidal ideation by aggregate groups and increasing support for SM youth in schools and communities.

## Introduction

Youth who identify as sexual minorities (SM; i.e., lesbian, gay, and bisexual) are significantly more likely to experience intimate partner violence (IPV) [1–4]. Experiencing IPV can lead to several adverse behavioral, physical, and mental health consequences. IPV can refer to either physical and/or sexual violence; physical IPV is defined as an intentional physical assault by a former or current partner and sexual IPV refers to forced sexual acts by a current or former partner [5].

### IPV among SM youth

Compared to their heterosexual peers, numerous studies reported that SM youth were significantly more likely to experience physical IPV [6–11], sexual IPV [6,8,11,12], and physical and/or sexual IPV [4,13–17]. Differences within SM youth have also been reported. Bisexual youth were more likely to report physical IPV [9], sexual IPV [2,8,18], and physical and/or sexual IPV [13,15] compared to their gay and lesbian identified peers. However, another study identified no differences in physical or sexual IPV among SM youth [17]. Findings indicate a need for awareness and further examination of IPV among vulnerable SM youth by identity.

SM youth who experienced IPV were more likely to express symptoms of depression and anxiety compared to heterosexual youth [17], due in part to lack of emotional support [19]. SM youth were also were more likely to engage in HIV-risk behaviors [20] such as engaging in sexual activity with more and older partners, have been pregnant, reported a sexually transmitted infection, have experienced sexual coercion, have had sex under the influence of drugs or alcohol, and were less likely to use contraception [7,14]. A study among female SM youth who were more likely to experience physical IPV reported that they had higher levels of depressive symptoms, lower academic achievement, and had a prior history of sexual activity [6].

### Suicidal ideation or attempts and IPV among SM youth

Suicide is the second leading cause of death among youth [21]. History of suicide attempts increase the likelihood of future greater self-harm and death by suicide [22,23]. In the year prior to the YRBS survey, 17.2% seriously considered, 13.6% of planned, 7.4% attempted, and 2.4% attempted a suicide resulting in treatment by a doctor [24]. A recent meta-analysis reported that suicidal ideation among youth strongly predicted future suicide attempts, however they also found that more than two-thirds of suicides occurred among youth without prior attempts [25]. Additionally, suicidal ideation and attempts were higher among females than males [24]. Suicidal ideation was highest among white youth compared to Black and Hispanic, yet suicide attempts where highest among Black youth [24]. While suicidal ideation rates declined among gay and lesbian youth between 1998 and 2003, bisexual male and female youth’s suicidal ideation increased between the same time period [26]. Despite decreased reports of suicidality, many studies found higher suicidal ideation and attempts among SM youth compared to their heterosexual peers regardless of gender [8,11,15,24,26–34] or when stratified by gender [35]. Bisexual youth reported the highest rates of suicidality compared to gay/lesbian and heterosexual youth [36,37].

Research examining differences between genders and SM identities have shown inconsistencies. One study reported female SM youth were more likely to report suicide attempts than their heterosexual peers; this relationship was not significant among male SM youth [38]. A later study found differences between genders such that suicide attempts were more likely among male SM youth than female SM youth [39]. Other results found that male SM youth were more likely to report suicidal ideation compared to female SM youth [8], while another reported that female SM youth were more likely to report suicidal ideation than male SM youth [3], and female bisexual youth reported more suicide attempts than male bisexual youth [40]. Gender differences among SM youth were only significant for suicidal ideation, but not planning or attempting suicide [3].

While research has examined some differences between genders, few studies have examined the relationship between experiencing a history of trauma, specifically physical and sexual IPV, and suicidal ideation and suicide attempts among SM youth. One study examining early life adversity and suicidality found that adverse child experiences including physical/sexual IPV, partially mediated the relationship between sexual orientation and suicidality [15]. In that particular study, researchers combined all early life adversity variables so it is unclear the role that IPV played in the relationship with suicidality. Another study examining a Midwestern population found the association between physical/sexual IPV and suicidal ideation/attempts was significantly stronger among SM youth compared to their heterosexual peers [41]. Additionally, SM youth who experienced physical/sexual IPV were significantly more likely to report suicidal ideation, planning, and attempts [3]. However, both studies combined physical and sexual IPV variables, and did not examine SM identity separately [3,41].

### Current study

The current study used the 2017 Youth Behavioral Risk Surveillance (YRBS) Survey, a nationally representative, diverse sample of youth to explore the relationship between physical IPV, sexual IPV, and suicidal ideation. We aimed to evaluate if SM identity enhanced the relationship between physical IPV and sexual IPV, and suicidal ideation and attempts. We hypothesized that 1) physical and sexual IPV would be associated with suicidal ideation and suicide attempts, 2) gay/lesbian and bisexual youth would be more likely to report suicidal ideation and suicide attempts compared to their heterosexual peers, and 3) the associations between IPV and suicidal ideation and suicide attempts would be stronger among both bisexual and gay/lesbian youth compared to their heterosexual peers, and stronger among bisexual youth compared to gay/lesbian youth. Additionally, we explored gender differences among any significant interactions, expecting bisexual female youth to have stronger associations compared to other SM youth.

## Methods and materials

The Institutional Review Board at the University of Texas approved the study. Consent was not obtained as this was a secondary data analysis using anonymous data.

### Population and sampling

Data for the present study was from the 2017 YRBS Survey [24]. The YRBS is a nationally representative, three-stage cluster sample survey design that provides comparable data from state, territorial, tribal, and large urban school districts. In addition to different regions, the YRBS sampling frame includes a variety of types of schools including both public and private. Detailed information on survey design is available elsewhere [24]. The University of Texas at Austin Institutional Review Board approved this secondary data analysis. The dataset used for the present study was retrieved after the CDC dropped 191 participants due to poor quality data for a total of 14,765 complete observations. The combined student and school response rate was 60% across a total of 144 schools [24].

### Measures

#### Demographic variables

The demographic variables measured were gender (male = 0, female = 1); age reported from 12 or younger to 18 or older in one-year increments and centered at 12 or younger; grade 9 through 12 centered at 9th grade; and race/ethnicity (white [reference], Asian/Pacific Islander, Black/African American, Hispanic, and other non-Hispanic). “Ungraded or other grade” responses on the grade variable were treated as missing values.

#### Sexual identity

Sexual identity was measured with a single question, “Which of the following best describes you?” Response options included “Heterosexual (straight)”, “Gay or lesbian”, “Bisexual”, and “Not sure”. The following groups were defined using the measure of sexual identity and gender: lesbian (female) youth, bisexual female youth, gay (male) youth, bisexual male youth, heterosexual female youth, and heterosexual male youth. “Not sure” responses were treated as missing values.

#### Physical and sexual IPV

Physical IPV was measured with the question, “During the past 12 months, how many times did someone you were dating or going out with physically hurt you on purpose?” Sexual IPV was measured with the question, “During the past 12 months, how many times did someone you were dating or going out with force you to do sexual things that you did not want to do?” Responses options for both questions included: 0 = I did not date or go out with anyone during the past 12 months, 0 = 0 times, 1 = 1 time, 2 = 2 or 3 times, 3 = 4 or 5 times, and 4 = 6 or more times. Variables were not combined in order to analyze the different types of IPV.

#### Dated in past 12 months

Respondents were assigned a zero if they selected “I did not date or go out with anyone during the past 12 months” to either the physical or sexual IPV variables above and were assigned one if they indicated that they had gone on at least one date on either the physical or sexual IPV variables.

#### Suicidal ideation

Suicidal ideation was measured with two items: 1) “During the past 12 months, did you ever seriously consider attempting suicide?” and 2) “During the past 12 months, did you make a plan about how you would attempt suicide?” An endorsement of either question was coded as 1. These two items were combined into one suicidal ideation variable as they both involve a thought process.

Suicide attempt. Suicide attempt was measured with two items: 1) “During the past 12 months, how many times did you actually attempt suicide?” and 2) “If you attempted suicide during the past 12 months, did any attempt result in an injury, poisoning, or overdose that had to be treated by a doctor or nurse?” Participants who reported attempting suicide 1 or more times or responded “yes” to the second question were coded as 1. These two items were combined to create one suicide attempt variable, as both items involve an action.

### Data analysis

YRBS data were sampled using a three-stage stratified cluster sample. Stratification was based on racial/ethnic concentration and metropolitan statistical area. Counties, groups of small, adjacent counties, or sub-areas of large counties were the primary sampling units. Weights were created using student demographics, nonresponse, and oversampling of minority students. Each of these design features were accounted for in the weighted data analyses. All data analyses were conducted using Stata 15.1. A total of four logistic regressions were fit for each outcome. Suicidal ideation was regressed on physical and sexual IPV main effects in the first two models. All models included the following covariates: dating in the past twelve months, age, gender, grade, and race/ethnicity. Interaction effects between sexual identity and physical and sexual IPV were added to the models. The above model sequence was repeated for suicide attempts. Significant interactions were probed by comparing the predicted values of bisexual v. heterosexual, gay/lesbian v. heterosexual, and bisexual v. gay/lesbian at low (0 times), medium (2 or 3 times), and high (6 or more times) levels of the IPV variables. Adjusted odds ratios (AOR) and 95% confidence intervals (CI) are presented.

Multiple imputation by chained equations (MICE) was used to replace missing values. Imputed data were generated using the other variables in the data to repeatedly update in models that imputed values for a putative distribution (e.g., multinomial). Race/ethnicity and sexual identity were imputed using a multinomial model; gender, dating in past twelve months, suicidal ideation, and suicide attempt were imputed using a logistic regression model, and the remainder of the variables were imputed using a linear regression. Twenty data sets with imputed values were created. Analysis of imputed data was performed separately on each data set containing imputed data; the model parameters and standard errors were combined across the twenty models in a final set of model parameters using Rubin’s rules [42]. We reported the relative increases in variance (RVI) and the fraction of missing information (FMI) for each model. This respectively assessed the proportional increase in sampling variance due to missing information and the proportion of sampling variance due to missing data.

## Results

### Population characteristics

Demographics, prevalence of physical and sexual IPV, and reported suicidal ideation and suicide attempts are presented in Table 1.

**Table 1:** Population characteristics of high school youth in the U.S.

Variables		Total % (95% CI)	Males % (95% CI)	Females % (95% CI)
Gender		--	49.3 (46.7, 51.9)	50.7 (48.1, 53.3)
Grade				
	9^th^	27.3 (25.6, 29.0)	27.7 (26.0, 29.4)	26.9 (25.1, 28.9)
	10^th^	25.7 (24.6, 26.8)	25.7 (24.0, 27.4)	25.7 (24.6, 26.9)
	11^th^	23.9 (23.3, 24.6)	23.9 (22.7, 25.1)	24.0 (23.0, 25.0)
	12^th^	23.1 (22.0, 24.2)	22.8 (21.4, 24.3)	23.4 (21.9, 24.9)
Age				
	12 or Younger	0.3 (0.2, 0.6)	0.5 (0.3, 0.8)	0.2 (0.1, 0.4)
	13	0.1 (0.1, 0.2)	0.1 (0.0, 0.2)	0.1 (0.0, 0.2)
	14	11.6 (10.5, 12.9)	10.7 (9.5, 12.0)	12.5 (11.0, 14.3)
	15	24.9 (23.7, 26.2)	25.4 (24.1, 26.8)	25.0 (23.5, 26.7)
	16	25.4 (24.1, 26.8)	25.0 (23.5, 26.7)	25.8 (24.1, 27.5)
	17	24.2 (22.9, 25.6)	24.5 (23.1, 26.0)	24.0 (22.2, 25.9)
	18 years or older	13.3 (12.2, 14.6)	14.3 (12.8, 15.8)	12.5 (11.1, 14.0)
Race/Ethnicity				
	Asian/Pacific Islander	4.1 (3.1, 5.4)	4.4 (3.2, 6.0)	4.3 (3.3, 5.5)
	Black/African American	13.4 (11.0, 16.3)	13.6 (11.0, 16.7)	13.3 (10.9, 16.1)
	White	53.5 (48.4, 58.5)	52.6 (47.6, 57.5)	54.3 (48.3, 60.2)
	Hispanic	22.8 (19.2, 26.9)	23.8 (20.3, 27.8)	21.9 (18.0, 26.4)
	Other (non-Hispanic)	6.0 (5.2, 6.9)	5.9 (4.9, 7.1)	6.1 (5.3, 7.0)
Sexual Identity				
	Heterosexual	89.3 (88.0, 90.4)	94.7 (93.3, 95.8)	83.7 (81.8, 85.5)
	Gay/Lesbian	2.4 (2.0, 3.0)	2.4 (1.7, 3.4)	2.5 (2.0, 3.0)
	Bisexual	8.3 (7.4, 9.3)	2.9 (2.3, 3.6)	13.8 (12.2, 15.7)
Dated Past 12 Months				
	No	31.0 (28.5, 33.7)	32.4 (29.5, 35.3)	29.7 (26.9, 32.6)
	Yes	69.0 (66.3, 71.5)	67.6 (64.7, 70.5)	70.3 (67.4, 73.1)
Physical IPV				
	0 times	94.6 (93.9, 95.2)	95.6 (94.9, 96.1)	93.6 (92.5, 94.5)
	1 time	2.1 (1.8, 2.5)	1.5 (1.3, 1.8)	2.8 (2.3, 3.4)
	2 or 3 times	1.7 (1.4, 2.0)	1.2 (0.9, 1.7)	2.1 (1.7, 2.7)
	4 or 5 times	0.6 (0.0, 0.8)	0.5 (0.3, 0.8)	0.6 (0.4, 0.9)
	6 or more times	1.0 (0.8, 1.3)	1.2 (0.9, 1.6)	0.9 (0.7, 1.2)
Sexual IPV				
	0 times	95.4 (94.8, 95.9)	98.1 (97.7, 98.5)	92.6 (91.5, 93.5)
	1 time	2.2 (2.0, 2.5)	0.7 (0.5, 1.0)	3.8 (3.4, 4.2)
	2 or 3 times	1.4 (1.1, 1.8)	0.3 (0.2, 0.5)	2.6 (1.9, 3.4)
	4 or 5 times	0.3 (0.2, 0.4)	0.2 (0.0, 0.5)	0.3 (0.2, 0.5)
	6 or more times	0.7 (0.5, 0.9)	0.6 (0.4, 0.9)	0.8 (0.5, 1.0)
Suicidal Ideation				
	No	80.1 (78.8, 81.2)	85.6 (84.3, 86.8)	74.7 (72.0, 77.2)
	Yes	19.9 (18.8, 21.2)	14.4 (13.2, 15.7)	25.3 (22.8, 28.0)
Suicide Attempt				
	No	92.7 (91.7, 93.6)	94.9 (94.0, 95.7)	90.7 (88.8, 92.3)
	Yes	7.3 (6.4, 8.3)	5.1 (4.3, 6.1)	9.3 (7.7, 11.2)

Weighted percentages and 95% confidence intervals are presented.

### Physical IPV

Weighted logistic regressions of sexual identity and physical IPV associated with suicidal ideation and suicide attempts are presented in Table 2. Female youth had approximately 50% greater odds of reporting suicidal ideation (AOR = 1.64, 95% CI: 1.40, 1.92, *p* < 0.001) and suicide attempts (AOR = 1.50, 95% CI: 1.16, 1.96, *p* < 0.001). Asian/Pacific Islander and other (non-Hispanic) youth were significantly more likely to report suicidal ideation (AOR = 1.38, 95% CI: 1.05, 1.81, *p* = 0.022; and AOR = 1.47, 95% CI: 1.20, 1.81, *p* < 0.001, respectively) compared to white peers. Black/African American and other (non-Hispanic) youth were significantly more likely to report suicide attempts (AOR = 1.48, 95% CI: 1.06, 2.06, *p* = 0.024; and AOR = 1.65, 95% CI: 1.23, 2.21, *p* = 0.002, respectively) compared to white peers. Bisexual youth had over five times greater odds of reporting suicidal ideation (AOR = 5.12, 95% CI: 4.16, 6.30, *p* < 0.001) and four times greater odds to report attempting suicide (AOR = 4.08, 95% CI: 3.01, 5.55, *p* < 0.001) compared to their heterosexual peers. Gay and lesbian youth had almost five times greater odds to report suicidal ideation (AOR = 4.62, 95% CI: 3.24, 6.59, *p* < 0.001) and over three times greater odds to report attempting suicide (AOR = 3.52, 95% CI: 2.13, 5.81, *p* < 0.001) compared to their heterosexual peers. While controlling for demographics and SM identity, youth who experienced physical IPV had almost two times greater odds of reporting suicidal ideation and attempting suicide (AOR = 1.69, 95% CI: 1.55, 1.86, *p* < 0.001; AOR = 1.89, 95% CI: 1.70, 2.09, *p* < 0.001, respectively) compared to their peers who did not experience physical IPV. There was no significant interaction between SM identity and physical IPV, and the association with suicide attempt; yet the interaction was significant for suicidal ideation.

**Table 2:** Association between physical IPV and suicidal ideation, and suicide attempt with moderation affects by sexual identity among high school youth in the US.

Variables		Suicidal Ideation			Suicidal Ideation Interaction		
		AOR (SE)	95% CI	*p*	AOR (SE)	95% CI	*p*
Did date (not date = ref)		**1.12 (0.06)**	**1.00, 1.25**	**0.046**	**1.12 (0.06)**	**1.00, 1.26**	**0.045**
Race/Ethnicity (white = ref)							
	Asian/Pacific Islander	**1.38 (0.18)**	**1.05, 1.81**	**0.022**	**1.38 (0.18)**	**1.05, 1.81**	**0.023**
	Black/African American	0.85 (0.09)	0.69, 1.04	0.107	0.85 (0.09)	0.69, 1.04	0.112
	Hispanic	0.98 (0.09)	0.81, 1.19	0.847	0.98 (0.09)	0.81, 1.19	0.867
	Other, non-Hispanic	**1.47 (0.15)**	**1.20, 1.81**	**0.001**	**1.47 (0.15)**	**1.19, 1.80**	**0.001**
Grade		0.98 (0.05)	0.88, 1.08	0.613	0.98 (0.05)	0.89, 1.07	0.599
Age		1.03 (0.05)	0.93, 1.13	0.599	1.02 (0.05)	0.93, 1.13	0.602
Female (male = ref)		**1.64 (0.13)**	**1.40, 1.92**	**< 0.001**	**1.64 (0.13)**	**1.40, 1.93**	**< 0.001**
Sexual Identity (heterosexual = ref)							
	Bisexual	**5.12 (0.52)**	**4.16, 6.30**	**< 0.001**	**5.48 (0.54)**	**4.48, 6.71**	**< 0.001**
	Gay/Lesbian	**4.62 (0.80)**	**3.24, 6.59**	**< 0.001**	**4.88 (0.87)**	**3.40, 7.02**	**< 0.001**
Physical IPV		**1.69 (0.08)**	**1.55, 1.86**	**< 0.001**	**1.81 (0.09)**	**1.65, 1.99**	**< 0.001**
Sexual Identity x Physical IPV (heterosexual = ref)							
	Bisexual	**--**	**--**	**--**	**0.71 (0.09)**	**0.55, 0.92**	**0.011**
	Gay/Lesbian	**--**	**--**	**--**	0.79 (0.14)	0.54, 1.14	0.198
		RVI = 0.09, FMI = 0.13			RVI = 0.11, FMI = 0.15		
		Suicidal Attempt			Suicidal Attempt Interaction		
		AOR (SE)	95% CI	*p*	AOR (SE)	95% CI	*p*
Did date (not date = ref)		**1.85 (0.23)**	**1.44, 2.38**	**< 0.001**	**1.85 (0.23)**	**1.44, 2.38**	**< 0.001**
Race/Ethnicity (white = ref)							
	Asian/Pacific Islander	1.38 (0.46)	0.71, 2.71	0.332	1.38 (0.46)	0.70, 2.70	0.339
	Black/African American	**1.48 (0.24)**	**1.06, 2.06**	**0.024**	**1.48 (0.24)**	**1.06, 2.07**	**0.022**
	Hispanic	1.32 (0.19)	0.98, 1.77	0.060	1.33 (0.19)	0.99, 1.77	0.056
	Other, non-Hispanic	**1.65 (0.24)**	**1.23, 2.21**	**0.002**	**1.63 (0.23)**	**1.21, 2.28**	**0.002**
Grade		0.92 (0.10)	0.74, 1.14	0.420	0.91 (0.09)	0.74, 1.13	0.395
Age		0.91 (0.10)	0.75, 1.11	0.358	0.92 (0.09)	0.75, 1.11	0.359
Female (male = ref)		**1.50 (0.88)**	**1.16, 1.96**	**0.003**	**1.51 (0.20)**	**1.16, 2.00**	**0.004**
Sexual Identity (heterosexual = ref)							
	Bisexual	**4.08 (0.61)**	**3.01, 5.55**	**< 0.001**	**4.42 (0.73)**	**3.15, 6.20**	**< 0.001**
	Gay/Lesbian	**3.52 (0.86)**	**2.13, 5.81**	**< 0.001**	**3.51 (0.93)**	**2.04, 6.04**	**< 0.001**
Physical IPV		**1.89 (0.09)**	**1.70, 2.09**	**< 0.001**	**1.10 (0.12)**	**1.75, 2.23**	**< 0.001**
Sexual Identity x Physical IPV (heterosexual = ref)							
	Bisexual	**--**	**--**	**--**	0.81 (0.12)	0.60, 1.08	0.144
	Gay/Lesbian	**--**	**--**	**--**	0.99 (0.19)	0.67, 1.46	0.961
		RVI = 0.29, FMI = 0.25			RVI = 0.31, FMI = 0.24		

Ref = reference group. RVI = relative increase in variance; FMI = fraction of missing information. Bolded values: *p* < 0.05. Models were weighted were based on student demographics, nonresponse, and oversampling of minority students (N = 14,765). Missing data was accounted for using multiple imputations (n = 20).

In light of the significant bisexual X physical IPV interaction (t [30] = -2.72, *p* = 0.011), we decomposed the SM X physical IPV interaction to examine SM differences at low, medium, and high levels of physical IPV. At zero instances of physical IPV, gay/lesbian respondents were significantly more likely than heterosexual respondents to have suicidal ideation (t [29] = 8.94, *p* < 0.001) as were bisexual respondents (t [30] = 17.20, *p* < 0.001); gay/lesbian and bisexual respondents did not differ (t [30] = 0.55, *p* = .586). At two to three instances of physical IPV, gay/lesbian respondents were significantly more likely than heterosexual respondents to report suicidal ideation (t [29] = 3.18, *p* = 0.004) as were bisexual respondents (t [30] = 3.87, *p* < 0.001); gay/lesbian and bisexual respondents did not differ (t [30] = -0.24, *p* = .811). At six or more instances of physical IPV, gay/lesbian respondents did not differ from heterosexual respondents in suicidal ideation (t [29] = 0.91, *p* = 0.369), nor did bisexual and heterosexual respondents (t [30] = 0.64, *p* = 0.525); gay/lesbian and bisexual respondents did not differ (t [30] = -0.40, *p* = .692). Additional analyses revealed no interaction between SM, physical IPV, and gender (*p* > 0.05).

### Sexual IPV

Weighted logistic regressions of SM and sexual IPV associated with suicidal ideation and suicide attempts are presented in Table 3. Results for female youth and SM youth are similar to those reported above such that females were significantly more likely to report suicidal ideation and attempts. Additionally, Asian/Pacific Islander and other (non-Hispanic) youth were significantly more likely to report suicidal ideation, and Black/African American and other (non-Hispanic) youth were more likely to report suicide attempts, compared to their white peers. Youth who experienced sexual IPV had almost two times greater odds to report suicidal ideation (AOR = 1.87, 95% CI: 1.61, 2.18, *p* < 0.001) and over two times greater odds to report attempting suicide (AOR = 2.08, 95% CI: 1.87, 2.32, *p* < 0.001) compared to their peers who did not experience sexual IPV. The interaction between SM identity and sexual IPV was not significant for suicidal ideation or suicide attempt.

**Table 3:** Association between sexual IPV and suicidal ideation, and suicide attempt with moderation affects by sexual identity among high school youth in the US.

		Suicidal Ideation			Suicidal Ideation Interaction		
		AOR (SE)	95% CI	*p*	AOR (SE)	95% CI	*p*
Did date (not date = ref)		1.12 (0.06)	1.00, 1.25	0.057	1.12 (0.06)	1.00, 1.26	0.056
Race/Ethnicity (white = ref)							
	Asian/Pacific Islander	**1.37 (0.20)**	**1.03, 1.83**	**0.032**	**1.37 (0.20)**	**1.03, 1.84**	**0.033**
	Black/African American	0.90 (0.09)	0.74, 1.09	0.277	0.90 (0.08)	0.74, 1.09	0.271
	Hispanic	0.99 (0.09)	0.82, 1.20	0.924	0.99 (0.09)	0.82, 1.20	0.927
	Other, Non-Hispanic	**1.50 (0.16)**	**1.22, 1.86**	**< 0.001**	**1.50 (0.16)**	**1.22, 1.85**	**< 0.001**
Grade		0.98 (0.05)	0.88, 1.09	0.691	0.98 (0.05)	0.89, 1.08	0.686
Age		1.03 (0.05)	0.93, 1.14	0.551	1.03 (0.05)	0.93, 1.13	0.547
Female (male = ref)		**1.56 (0.12)**	**1.33, 1.82**	**< 0.001**	**1.55 (0.12)**	**1.33, 1.81**	**< 0.001**
Sexual Identity (heterosexual = ref)							
	Bisexual	**5.14 (0.51)**	**4.20, 6.30**	**< 0.001**	**5.25 (0.53)**	**4.27, 6.44**	**< 0.001**
	Gay/Lesbian	**4.72 (0.82)**	**3.31, 6.74**	**< 0.001**	**4.78 (0.82)**	**3.36, 6.79**	**< 0.001**
Sexual IPV		**1.87 (0.14)**	**1.61, 2.18**	**< 0.001**	**1.91 (0.16)**	**1.62, 2.27**	**< 0.001**
Sexual Identity x Sexual IPV (heterosexual = ref)							
	Bisexual	**--**	**--**	**--**	0.88 (0.15)	0.62, 1.26	0.479
	Gay/Lesbian	**--**	**--**	**--**	0.93 (0.29)	0.49, 1.78	0.821
		RVI = .09, FMI = .13			RVI = .10, FMI = .15		
		Suicidal Attempt			Suicidal Attempt Interaction		
		AOR (SE)	95% CI	*p*	AOR (SE)	95% CI	*p*
Did date (not date = ref)		**1.85 (0.23)**	**1.44, 2.28**	**< 0.001**	**1.85 (0.23)**	**1.44, 2.38**	**< 0.001**
Race/Ethnicity (white = ref)							
	Asian/Pacific Islander	1.38 (0.48)	0.67, 2.81	0.370	1.37 (0.48)	0.66, 2.81	0.385
	Black/African American	**1.66 (0.26)**	**1.21, 2.28**	**0.003**	**1.65 (0.26)**	**1.20, 2.26**	**0.003**
	Hispanic	1.34 (0.20)	0.99, 1.81	0.056	1.34 (0.20)	1.00, 1.81	0.053
	Other, Non-Hispanic	**1.69 (0.25)**	**1.25, 2.30**	**0.001**	**1.69 (0.25)**	**1.24, 2.29**	**0.001**
Grade		0.92 (0.10)	0.73, 1.16	0.480	0.92 (0.10)	0.73, 1.16	0.449
Age		0.93 (0.10)	0.75, 1.15	0.468	0.93 (0.10)	0.75, 1.16	0.512
Female (male = ref)		**1.39 (0.17)**	**1.08, 1.80**	**0.012**	**1.38 (0.17)**	**1.07, 1.79**	**0.015**
Sexual Identity (heterosexual = ref)							
	Bisexual	**4.13 (0.60)**	**3.07, 5.56**	**< 0.001**	**4.48 (0.68)**	**3.23, 6.12**	**< 0.001**
	Gay/Lesbian	**3.71 (0.86)**	**2.31, 5.97**	**< 0.001**	**3.59 (0.89)**	**2.15, 5.98**	**< 0.001**
Sexual IPV		**2.08 (0.11)**	**1.87, 2.32**	**< 0.001**	**2.19 (0.14)**	**1.92, 2.49**	**< 0.001**
Sexual Identity x Sexual IPV (heterosexual = ref)							
	Bisexual	**--**	**--**	**--**	0.78 (0.10)	0.59, 1.01	0.063
	Gay/Lesbian	**--**	**--**	**--**	1.12 (0.29)	0.66, 1.90	0.671
		RVI = 0.25, FMI = 0.24			RVI = 0.26, FMI = 0.22		

Ref = reference group. RVI = relative increase in variance; FMI = fraction of missing information. Bolded values: *p* < 0.05. Models were weighted were based on student demographics, nonresponse, and oversampling of minority students (N = 14,765). Missing data was accounted for using multiple imputations (n = 20).

## Discussion

The present study evaluated the relationship between IPV and suicidal ideation and attempts, SM identity separately with suicidal ideation and attempts, and then determined if the association between IPV and suicidal ideation and attempts were attenuated by SM identities. As hypothesized, youth who experienced physical or sexual IPV were significantly more likely to report suicidal ideation and suicide attempts. These results are supported by previous studies [35,43–47]. Studies reported associations between IPV and mental health symptoms, which may lead to suicidal thoughts or actions [17,35,46]. To better understand the direction and relationships of these associations between variables, longitudinal studies are needed.

In the present study, regardless of experiencing physical or sexual IPV, Asian/Pacific Islander youth were more likely to report suicidal ideation; Black/African American youth were more likely to report suicide attempts; and other (non-Hispanic) youth were more likely to report both suicidal ideation and attempts, compared to their white peers. Supporting previous studies, SM youth were significantly more likely to report suicidal ideation and suicide attempts compared to heterosexual peers [3,8,11,15,26–34], and bisexual youth [26,40]. The relationship between minority status and suicidal ideation or attempts may best be understood within the minority stress model [48,49]. Minority social groups, such as racial/ethnic and/or SM-identity, may experience increased stress due to their minority status [49].

Minority stress stemming from racism, homophobia, and poverty has been found to be associated with suicidal ideation, depression, and anxiety among Latino MSM [50]. The minority stress model suggests that stress related to one’s minority status has negative consequences on an individuals’ health and well-being [48]. Sexual and gender minorities experience stress via experiences of prejudice, stigma, and chronic discrimination, which may influence sexual and drug use behaviors and negatively affect health [49]. SM youth may be exposed to prolonged stigma associated with their sexual identity, which can lead to negative opinions of themselves or other SM individuals, concealing their identity, and expecting future discrimination and ostracization [49,51]. These negative outcomes associated with minority stress are also associated with increased risk of IPV [4]. Research has shown homonegativity and sexual identity concealment were positively associated with perpetration and experience of IPV [52–55].

While the YRBS does not include measures of minority stress, the greater odds of physical IPV and suicidal ideation reported by bisexual participants in this study may reflect experiences of anticipated or internalized homonegativity experienced by SM youth. This is of particular concern among bisexual youth who tend to experience worse health outcomes than gay/lesbian and heterosexual youth [17,40].

Bisexual identity modified the relationship between physical IPV and suicidal ideation. Bisexual youth were significantly more likely than their heterosexual peers to report suicidal ideation at low and medium instances of physical IPV, but there was no significant difference at high levels of IPV. This relationship was not attenuated by gender. There was no difference between bisexual and gay/lesbian youth. Differences between bisexual and heterosexual youth at low and medium levels of IPV may be explained by the stress experienced from identifying as bisexual. Bisexual youth may be exposed to greater stigma from both heterosexual and gay/lesbian individuals [37]. While SM youth may become engaged in relationships that involve IPV due to low self-worth or feeling helpless in finding a romantic partner [4], this may be increasingly likely among bisexual youth from lack of inclusion from both heterosexual and gay/lesbian individuals [37]. As experiences of IPV increased to a high level, the lack of difference found between bisexual and heterosexual may be due to the severe negative effects of IPV, such as depression and post-traumatic stress symptoms [35,46].

This research is of concern to educators and school administrators as findings note implementing evidence-based policies and programs that promote inclusivity and positive sexual and gender identity development for SM youth are highly regarded. Schools with established gay-straight alliances and policies prohibiting homophobia, SM youth had fewer suicidal thoughts and attempts [56].

### Limitations

Although the 2017 YRBS represents a national sample of youth, there are limitations that should be considered. First, the data does not account for students not enrolled or attending school. Second, underreporting of physical, sexual IPV, suicidal ideation or identifying as SM youth may have occurred, particularly among males [57–61]. Third, we were unable to include youth who identified as transgender as this data was not collected in the 2017 YRBS. Fourth, the YRBS does not collect data regarding SM-related stress, therefore not allowing researchers to integrate this work into our assessments. Finally, the data are anonymous and cross sectional, thus the causal nature of the relationship between experiences of violence and suicidal ideation or attempts cannot be determined.

### Future research

The present research findings demonstrate the need for attention to the high reported suicidal ideation and suicide attempts among SM youth who experience physical or sexual IPV. Research has shown positive attributes to SM support and ally groups [62]. SM youth reported that school- or community-based SM groups were resources for youth who experienced IPV [18]. Schools with established gay-straight alliances and policies prohibiting homophobia had fewer suicidal thoughts and attempts [56], thus increasing the presence of such organizations in schools and the community may be beneficial for SM youth.

These findings support the possibility of utilizing social network theory to teach heterosexual and SM youth how to engage with SM youth who disclose IPV or suicidal thoughts. Any IPV policies or program should include violence among partners, regardless of the gender of each partner so that students are not excluded, to change the heteronormative atmosphere [17,33]. With physical and sexual IPV, and suicidal ideation leading to medical visits, healthcare professionals in all sectors must be trained to interact, address, and provide resources for individuals of all sexual and gender minorities who experience IPV [19]. Schools and medical providers should consider adopting policies so that all resources, paperwork, and announcements are gender-neutral. Finally, future research is needed to assess the extent to which minority stress factors may contribute to IPV and suicidal ideation among SM youth. These may be useful factors to include in future iterations of the YRBS survey to better understand the extent of these stressors experienced by youth and identify potential protective factors.

## Conclusions

While the YRBS data has its limitations, the data serves as an excellent source for examining the relationship between high-risk variables and underrepresented groups. The data provided rich knowledge about historically under-represented groups and fills a gap in research examining high-risk variables by utilizing a diverse, nationally representative sample to examine sensitive information across under-representative groups. The present analysis suggests further attention be drawn to individuals experiencing IPV as it is indicative of future self-harming behaviors. Furthermore, findings suggest research continue to focus on SM youth, particularly by identity, to obtain more information on physical and mental health outcomes.
